# Artisanal Household Milk Pasteurization Is Not a Determining Factor in Structuring the Microbial Communities of Labneh Ambaris: A Pilot Study

**DOI:** 10.3390/foods11233874

**Published:** 2022-12-01

**Authors:** Reine Abi Khalil, Christel Couderc, Sophie Yvon, Gwenaelle Jard, Delphine Sicard, Frédéric Bigey, Rabih El Rammouz, Pierre Abi Nakhoul, Hélène Eutamène, Hélène Tormo, Marie-José Ayoub

**Affiliations:** 1 Department of Food Sciences and Technologies, Faculty of Agricultural and Veterinary Sciences, Lebanese University, Beirut 14-6573, Lebanon; 2 Département Sciences de l’agroalimentaire et de la Nutrition, Université de Toulouse, INP EI-Purpan, 75 voie du TOEIC, BP 57611, CEDEX 3, F-31076 Toulouse, France; 3 Toxalim, UMR 1331, INRA, INP-ENVT, INP-PURPAN, Université de Toulouse, 31027 Toulouse, France; 4 INRAE, Institute Agro Montpellier, SPO, University Montpellier, 34060 Montpellier, France; 5 Department of Animal Production, Faculty of Agricultural and Veterinary Sciences, Lebanese University, Beirut 14-6573, Lebanon

**Keywords:** labneh Ambaris, raw milk, artisanal pasteurization, microbial diversity, DNA metabarcoding

## Abstract

Labneh Ambaris is a traditional Lebanese dairy product traditionally made using raw goat’s milk in earthenware jars, but recently the use of artisanally pasteurized milk was introduced for safety reasons. In this study, 12 samples of labneh Ambaris were studied, six made using raw goat’s milk and six others using artisanally pasteurized goat’s milk. These samples were collected during fermentation and their microbial compositions were analyzed. The 16S V3–V4 and the ITS2 regions of the rDNA were sequenced by DNA metabarcoding analyses for the identification and comparison of bacterial and fungal communities, respectively. The samples had high microbial diversity but differences in samples microbiota were unrelated to whether or not milk was pasteurized. The samples were consequently clustered on the basis of their dominant bacterial or fungal species, regardless of the milk used. Concerning bacterial communities, samples were clustered into 3 groups, one with a higher abundance of *Lactobacillus helveticus*, another with *Lactobacillus kefiranofaciens* as the dominant bacterial species, and the third with *Lentilactobacillus sp.* as the most abundant species. Species belonging to the *Enterobacteriaceae* family were detected in higher abundance in all raw milk samples than in artisanally pasteurized milk samples. As for fungal communities, the samples were clustered into two groups, one dominated by *Geotrichum candidum* and the other by *Pichia kudriavzevii*.

## 1. Introduction

Traditional dairy products have been produced around the world for thousands of years. Their quality and characteristics are determined by several factors closely related to their terroir of origin, such as the climatic conditions, the type of animal feed as well as the ancestral manufacturing practices. They are much appreciated by consumers due to their unique tastes and aromas. Unlike industrial dairy products, traditional ones are usually produced in small dairies or at household level using raw milk, which results in high microbial diversity and the preservation of indigenous milk microbiota [1,2,3]. However, the safety of raw milk dairy products and their potential risks to humans upon consumption have been extensively studied, and several diseases or outbreaks related to the consumption of dairy products contaminated by pathogens have been reported [4,5].

Pasteurization is widely used in the production of dairy products. Its main purpose is to improve milk hygiene by the inactivation of pathogenic microorganisms that could be present and to lower the overall microbial load [6,7]. It was determined in previous studies that the indigenous microbiota of raw milks contribute to the sensory qualities of raw milk dairy products, providing them with more pronounced and unique sensory characteristics [3,8]. On the other hand, it is reported that dairy products made with pasteurized milk can have lower aromatic complexity due to their lower microbial diversity and richness [9]. 

To study microbial diversity, culture-dependent microbiological methods are traditionally used. These methods use selective and nutritive growth media to enumerate and isolate microorganisms. While this is useful for tracking viable and cultivable microorganisms, culture-dependent methods have limited abilities to detect rare and difficult-to-culture species [10,11]. In recent years, culture-independent methods have become popular in the study of microbial diversity. These techniques rely on DNA analyses based on molecular methods with high throughput sequencing (HTS), such as DNA metabarcoding. The latter uses universally targeted molecular markers (barcodes) shared by various taxonomic groups (16S for bacteria and ITS2 for fungi). HTS methods deliver deeper information on both dominant and rare species which gives a more global vision of the microbial diversity in food matrices compared to culture-dependent methods [10,11,12]. Many recent studies have used this method to characterize and identify microbial populations in fermented milks [13,14,15,16].

Labneh Ambaris is a traditional fermented milk product made in rural areas in Lebanon. It is typically produced by spontaneous fermentation of raw goat’s milk with coarse salt in special earthenware jars. A distinguishing feature of its production process is the regular addition of milk and salt after each coagulation and whey drainage until the jars are full of coagulum [17,18]. Due to food safety concerns, some producers have been artisanally pasteurizing the milk in their households for their productions. To our knowledge, no published data comparing the microbiota (both bacteria and fungi) of labneh Ambaris produced using raw or pasteurized goat’s milk using metabarcoding analysis are available in the literature. Therefore, the aim of this work was to study the impact of artisanal household milk pasteurization using a DNA metabarcoding approach to analyze the microbial composition and diversity of labneh Ambaris samples produced in 12 different households using raw or artisanally pasteurized goat’s milk.

## 2. Materials and Methods

### 2.1. Sampling

Twelve labneh Ambaris samples were each collected from a producer in Lebanon. All of them were made by spontaneous fermentations in earthenware jars without the addition of starters. Six were made using raw goat’s milk and the other six using pasteurized goat’s milk. Artisanal household pasteurization was applied by the producers, in which milk was heated until it reached boiling temperature, held for a maximum of 5 min, and then allowed to cool at room temperature. Labneh Ambaris productions were carried out at room temperature. Samples were collected from their production jars during the mid-fermentation stage (at minimum, 2 months after the beginning of fermentation) and they were ready to be consumed at this stage. They were frozen at −20 °C to be later submitted for DNA metabarcoding sequencing and physicochemical analyses. Raw milk samples (RMSs) were codified as La-R1 to La-R6 and artisanally pasteurized milk samples (PMSs) were codified as La-P1 to La-P6.

### 2.2. pH Measruments

The pH values were recorded in triplicates at room temperature using a calibrated pH meter (HQ11 HD, HACH, Manchester, UK). An ANOVA statistical test was applied to the data grouped according to the milk type (RMS, PMS) using XLSTAT (Addinsoft, 2022, Paris, France).

### 2.3. DNA Extraction, Metabarcoding Sequencing and Bioinformatics Analyses

Total DNA was extracted using the DNeasy PowerFood Microbial Kit (Qiagen, Hilden, Germany). DNA concentrations were then determined using a nanodrop spectrophotometer (NanoDrop 2000/2000c, Thermo Scientific, Wilmington, DE, USA) and were adjusted to 10 ng/µL. Mock communities (a mix of equimolar DNA concentrations (10 ng/µL) of several known species) were added for bacterial and fungal sequencing. The bacterial mock was composed of *Lactiplantibacillus plantarum*, *Lentilactobacillus diolivorans*, *Lactobacillus delbrueckii*, *Lentilactobacillus parabuchneri*, *Lacticaseibacillus rhamnosus*, and *Lactococcus lactis*. Similarly, the fungal mock was composed of *Kluveromyces marxianus*, *Kazachstania exigua*, *Pichia kudriavzevii*, *Saccharomyces cerevisiae*, *Kluveromyces lactis*, *Rhodotorula mucilaginosa*, *Torulaspora delbrueckii*, *Pichia terricola*, *Candida parapsilosis*, and *Debaryomyces hansenii*. 

The bacterial and fungal diversities were evaluated by DNA metabarcoding sequencing of the amplified V3–V4 region of the 16S rDNA for bacteria (amplicon size of 426 bp) and the ITS2 region for fungi (amplicon sizes between 187 bp and 367 bp). For Illumina MiSeq sequencing, PCR conditions were used as described by von Gastrow et al. [19]. Briefly, the primers used to amplify the V3-V4 region were 16SV3 Forward (5′-TACGGRAGGCAGCAG-3′) and 16SV4 Reverse (5′-TACCAGGGTATCTAATCCT-3′), and the primers for the ITS2 region were ITS3 tagmix1 Forward (5′-CTAGACTCGTCATCGATGAAGAACGCAG-3′) and ITS4ngs Reverse (5′-TTCCTSCGCTTATTGATATGC-3′). Illumina tails were added to the primers along with frameshifts (sequences of 4 to 8 random nucleotides). The resulting PCR amplicons were then amplified in a second PCR to incorporate the dual-indexed adaptors for each sample. All amplicons were purified after each PCR, quantified and then pooled together to form the library. The mix was then sequenced by following an Illumina MiSeq protocol, generating 300 bp paired-end reads.

The resulting sequences were analyzed using two pipelines. The pre-processing steps were carried out using the DADA2 package version 1.14.1 in R program (version 3.6.1, R Core Team, Vienna, Austria). This resulted in amplicon sequence variant (ASV) tables. Taxonomic affiliations using the DAIRYdb database version 1.1.2 [20] for 16S rRNA and the UNITE database version 8.2 [21] for ITS2 sequences were determined using FROGS version 3.2.2 [22]. Multi-affiliations were checked using affiliation Explorer [23]. ASVs with abundances of less than 0.5% of all sequences were excluded from the analyses because they were not reproduced between runs, as described by Paës et al. [24]. ASV tables were then rarefied while creating the Phyloseq Object with FROGSSTAT Phyloseq Import Data (Galaxy Version 3.2.2) before assessing the samples diversities. Species diversity within each sample was estimated using α-diversity indices (richness and Shannon) using the FROGGSTAT Phyloseq Alpha Diversity tool (Galaxy Version 3.2.2). The species diversity between the RMS and PMS communities (β-diversity) was estimated by Bray–Curtis dissimilarity indices using the FROGGSTAT Phyloseq Beta Diversity tool (Galaxy Version 3.2.2). MANOVA was then performed using FROGSSTAT Phyloseq Multivariate Analysis of Variance (Galaxy Version 3.2.2) on both bacterial and fungal matrices to detect the existence of significant differences between communities based on the milk type (RMS, PMS) as the experimental variable. Hierarchical clustering of the 12 samples based on the Ward linkage method and Bray–Curtis indices was then applied and visualized using the FROGSSTAT Phyloseq Sample Clustering tool (Galaxy Version 3.2.2).

## 3. Results

### 3.1. pH

The details of the recorded pH values are shown in Table 1. All values were lower than four. No significant differences existed between the pH values of the raw milk samples (RMS group) and the artisanally pasteurized milk samples (PMS group), at a 95% confidence interval.

### 3.2. Bacterial Communities

**Figure 1:**
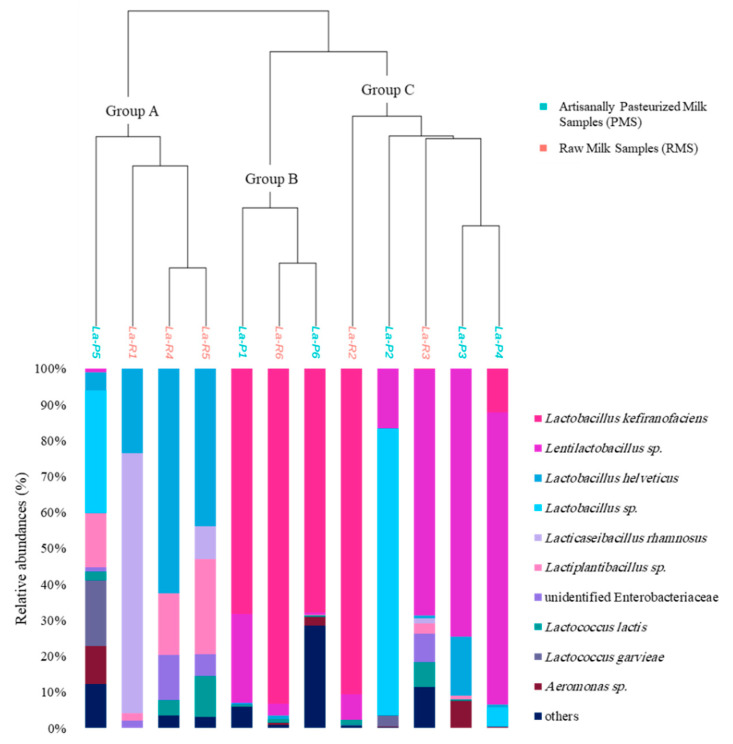
Analysis of bacterial communities in 12 labneh Ambaris samples (6 made with raw goat’s milk (RMSs) and 6 made with artisanally pasteurized goat’s milk (PMSs)) by DNA metabarcoding sequencing of the 16S rDNA V3-V4 region. The hierarchical ascendant classification (HAC) plot is based on Bray–Curtis dissimilarity matrices and the distribution of the major 10 bacterial species within each sample.

The 16S rDNA analysis resulted in the identification of 2 phyla, 6 families, 11 genera and 15 species. Some species could not be identified at the genus level, but they all belonged to the Enterobacteriaceae family. It was noticeable that these species were detected in all RMSs in higher abundances (minimum 0.06% in La-R6 up to 12.65% in La-R4, with three out of the six RMSs having >6% relative abundance) than in PMSs (ranging from not detected to a maximum of 1.18% in sample La-P5, with all PMSs ≤ 2%). No *Salmonella* sp., *Staphylococcus aureus*, *Listeria monocytogenes*, *Escherichia coli* or *Brucella* sp. were detected within the 12 samples. Species richness for the 12 samples was assessed using two alpha diversity indices. Based on the 21 ASVs remaining after filtering at 0.5% relative abundance, most samples had similar richness indices ranging between 10 and 13 ASVs representing 8 to 11 species, except for La-R1 (6 ASVs representing 4 species), and La-P6 (7 ASVs each representing a species). The richness within each sample was also assessed using the Shannon index, which showed that La-P5 (with a Shannon value of 1.97, the highest value among the samples) had relative abundances distributed between several bacterial species (Figure 1). The lowest values were recorded for La-R6 and La-R2 (Shannon indices of 0.38 and 0.41, respectively), both dominated by *L. kefiranofaciens* with more than 85% relative abundance. The details of the bacterial alpha diversity indices are shown in Table 1.

The MANOVA statistical analysis applied to Bray–Curtis dissimilarity indices showed no significant differences between bacterial communities according to the milk type used for production (RMS vs PMS). All samples were clustered into 3 groups by hierarchical ascendant classification (HAC) based on Bray–Curtis distance matrices using Ward’s linkage method (Figure 1). Clustering was independent of the milk heat treatment. The samples constituting the first group, Group A (La-P5, La-R1, La-R4, and La-R5), had *L. helveticus* and *Lactiplantibacillus* sp. in higher abundances within their compositions than other samples. The species *L. kefiranofaciens* was not detected in any of these samples and *Lentilactobacillus* sp. was detected at a very low relative abundance (0.92%) in one sample only, La-P5. Sample La-R1 was dominated by *Lacticaseibacillus rhamnosus* at 72.36% relative abundance. As for the second group (Group B), it included samples La-P1, La-R6, and La-P6 which had *L. kefiranofaciens* as the dominant bacterial species at more than 67% relative abundance within each sample. In Group C, samples La-R3, La-P3, and La-P4 were all dominated by *Lentilactobacillus* sp., which was present at more than 68% relative abundances. Sample La-P2 was included in the same group since it contained *Lentilactobacillus* sp. (16.53% relative abundance). Although the bacterial composition of sample La-R2 was closer to that of Group B, it was ordinated in a single branch within Group C. This may be explained by the presence of *L. garvieae* in La-R2 (0.1% relative abundance), as for the samples in Group C where its abundance ranged from 0.03% to 2.95%, noting that this species was not detected in any of the samples of Group B. The species *Lactococcus lactis* was detected in 11 out of the 12 samples with relative abundances ranging from 0.02% to 11.37%. *Lactiplantibacillus* sp. was detected in all 12 samples. The species *Lacticaseibacillus rhamnosus* was detected in four RMSs at abundances varying from 1.34% to 72.36%, whereas it was only detected in two PMSs at very low abundances (0.02% and 0.03%).

### 3.3. Fungal Communities

**Figure 2:**
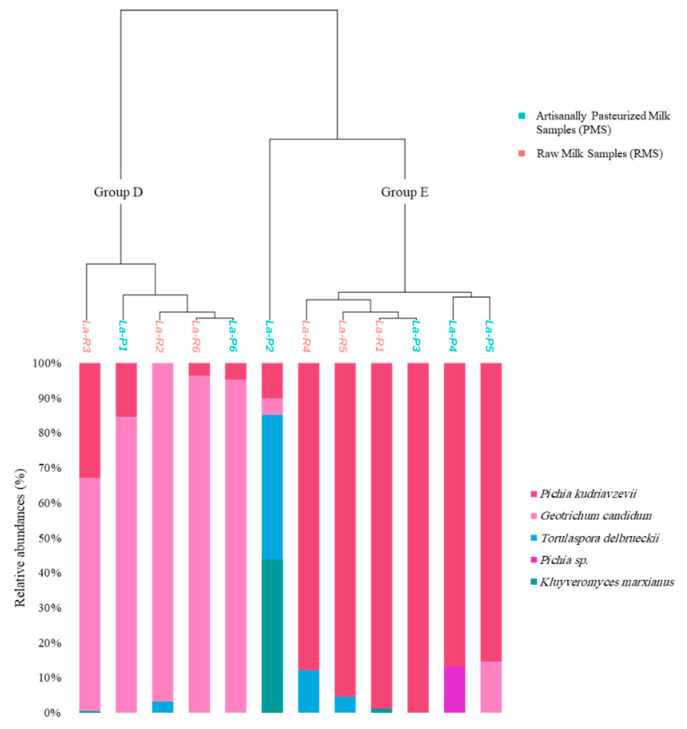
Analysis of fungal communities in 12 labneh Ambaris samples (6 made with raw goat’s milk (RMSs) and 6 made with artisanally pasteurized goat’s milk (PMSs)) by DNA metabarcoding sequencing of the ITS2 region. The hierarchical ascendant classification (HAC) plot is based on Bray–Curtis dissimilarity matrices and the distribution of the 5 remaining fungal species within each sample.

The ITS2 sequencing analysis resulted in the detection of one phylum, three families, four genera and five species. Independently of the type of milk used, less diversity existed concerning fungal compositions compared to bacterial compositions and it was clear that most of the samples were dominated by one of two fungal genera, *Geotrichum* or *Pichia*. Within the identified species, *Pichia kudriavzevii* and *Geotrichum candidum* were the most abundant, representing 49.13% and 48.12% of the total number of sequences, respectively. As shown in Figure 2, *G. candidum* and *P. kudriavzevii* were detected in all 12 samples at varying relative abundances. In addition, *T. delbrueckii* was detected in five samples (three RMSs and two PMSs) with relative abundances ranging from 0.02%% to 41.45%. *K. marxianus* was detected in three samples; the relative abundances were 0.46% and 1.19% in La-R3 and La-R1, respectively, and its relative abundance amounted to 43.75% in sample La-P2. Once the ASVs with abundances lower than 0.5% of the total sequences were discarded, only five fungal ASVs remained and the fungal species richness for the 12 samples was assessed using the richness and Shannon alpha diversity indices as it was done for bacteria. Based on the number of ASVs within each sample, the most diverse sample was La-P2, with a richness index of four representing four species within its composition, whereas the least diverse sample was La-P3 with only one remaining ASV detected, corresponding to *P. kudriavzevii*. The richness within each sample was also assessed using the Shannon index, whose value was highest in sample La-P2 (value 1.09) where relative abundances were distributed between three fungal species (Figure 2). The value of the Shannon index was lowest in La-P3 (value 0), which was dominated by one species, *P. kudriavzevii*, at 99.2% relative abundance. It was noticeable that sample La-R1 had a high richness index but a very low Shannon index (0.07), which can be explained by the dominance of *P. kudriavzevii* at 95.8% of relative abundance.

The MANOVA statistical analysis applied to the Bray–Curtis dissimilarity indices showed that there were no significant differences between fungal communities according to the milk heat treatment used for production (RMSs vs. PMSs). The samples were clustered into two main groups using a hierarchical ascendant classification (HAC) based on the Bray–Curtis distance matrix and Ward’s linkage method (Figure 2). The first group, Group D (La-R3, La-P1, La-R2, La-R6 and La-P6), was dominated by the species *G. candidum* at more than 65% relative abundance within each sample. The second group, Group E (La-R4, La-R5, La-R1, La-P3, La-P4, La-P5), was dominated by *P. kudriavzevii* at more than 83% relative abundance. The sample La-P2 was co-dominated by the species *K. marxianus* (43.75% relative abundance) and *T. delbrueckii* (41.45% relative abundance) and therefore was not clustered with any of the other two groups.

## 4. Discussion

Labneh Ambaris is a fermented milk product in which coagulation occurs mostly after the production of lactic acid from lactose fermentation by lactic acid bacteria. It is traditionally made with only raw goat’s milk and coarse salt. However, many households and producers have recently begun to replace raw milk with pasteurized milk, mostly due to food safety concerns. In this pilot study, the microbial compositions of 12 labneh Ambaris samples (six made using raw milk and six others using pasteurized milk) collected during the mid-fermentation stage were compared using DNA metabarcoding analyses to check whether artisanal milk pasteurization applied at the household level influences the microbial composition and diversity of Ambaris. Our preliminary findings revealed that the microbial compositions of labneh Ambaris were highly diverse, independently of the milk being artisanally pasteurized or raw. Our results also showed that *Enterobacteriaceae* species were present in all the samples studied, whether made of raw or pasteurized milk, although with varying abundances. *Dairy products made from pasteurized milk are usually inoculated with starter cultures to initiate fermentation after the native microbiota of the milk has been deactivated* [25]. *In our case, no starter cultures were added to our labneh Ambaris samples prepared with artisanally pasteurized milk (PMSs). Nonetheless, milk coagulation occurred at very low pH values similar to those of raw milk samples (RMSs)*.

Despite the fact that raw milk pasteurization reduces pathogens and indigenous bacterial and fungal loads [6,26,27], the fungal and bacterial communities of labneh Ambaris samples made from pasteurized and raw milk were largely comparable, although they were diverse. In contrast, the results of two recent studies demonstrated that cheeses (Gouda and Belgian soft-cheese) made with pasteurized milks had lower microbial diversity (richness) than ones made with raw milk [8,28]. Several factors may explain these contradictory observations. Indeed, the cheeses in these two studies were made under controlled industrial conditions and starter cultures were added to allow milk coagulation, whereas the labneh Ambaris samples in this study were artisanally made and the pasteurization parameters were not fully controlled. In addition, some microbial species can still survive the artisanal heat treatment since heat sensitivity varies with genus and species [26,29]. The initial microbial load of raw milk also plays a role in determining the remaining load of microorganisms after pasteurization [30]. *In many developing Middle Eastern countries including Lebanon, hand-milking is mainly practiced with small ruminants, which results in long milking times* [4]. *Furthermore, milk is kept at room temperature until milking is finished and only then it is transported and used, which could result in high initial microbial loads in the raw milk. Consequently, artisanal pasteurization practiced at the household level on raw milk with relatively high microbial loads could allow microorganisms to remain in the matrix* [2]. *To confirm this hypothesis, it would be interesting to enumerate microbial groups in milk that is handled that way before and after pasteurization is applied*.

On the other hand, the presence of microorganisms in the samples could be due to contaminations that occurred after heat treatments and that originated from the surrounding production environment [8]. Various studies have found that the microbial species that colonize production facilities and the air in the manufacturing areas have a significant impact on the microbial compositions of dairy products [31,32,33]. A study showed that the same yeast species were detected in cheese and in the environment of its production facility, although at varying abundances [34]. It would be interesting to identify the microbial populations that are present in the production environment to confirm this hypothesis.

Furthermore, the persistence of microorganisms from one production season to the following could inoculate the new productions and therefore explain the presence of the same major bacterial and yeast species within raw milk samples (RMSs) and artisanally pasteurized milk samples (PMSs). Microorganisms, especially non-starter lactic acid bacteria (NSLAB) that are dominant in labneh Ambaris, could be transferred from the dairy equipment used, mainly the earthenware jars, where they could have persisted from previous productions through biofilm formation [29,35]. These structured communities of self-preserved microorganisms could adhere to the inner surfaces of the earthenware jars and remain until the following season. Then, when the conditions are favorable for growth, the microbial cells previously encapsulated in their matrix would be dispersed into the freshly added milk [36]. Extracellular exopolysaccharides (EPS) are key components for the formation of biofilms. They have roles in cell-to-cell and cell-to-surface adhesions [37,38]. Several species have been described to produce EPS, such as *L. kefiranofaciens*, *L. kefiri*, *L. plantarum*, *L. rhamnosus*, *L. lactis* and *L. brevis* [39,40]; all of them found dominantly in all our labneh Ambaris samples. Studies have shown that inside wooden vats used for the production of Italian PDO cheeses, LAB biofilms were formed from the previous productions [41]. Such biofilms could thus be a vector of microorganisms and contribute to their dissemination and inoculation into the following productions [42]. It would consequently be interesting to verify if the same phenomenon takes place in earthenware jars.

*Enterobacteriaceae* species were found in all RMSs and were more abundant in RMSs than in PMSs. Raw milk used in the production of labneh Ambaris RMSs may harbor high bacterial loads of active *Enterobacteriaceae* species that could proliferate during production, as was recently shown during production of raw milk Ambaris [18]. In contrast, in PMSs, these microorganisms should have been deactivated after milk heating. However, these species have also been discovered in PMSs, suggesting that their presence could be the consequence of cross-contaminations. Indeed, during the manufacturing process milk is regularly added to the jars and there are several instances of human intervention; these are different factors that can contribute to the presence of these species [43]. Insufficient artisanal pasteurization and high initial loads could also be contributing factors as explained before. Nevertheless, we must raise the fact that the high abundances detected in RMSs with our methodological approach do not imply that the *Enterobacteriaceae* species were viable in our samples; therefore, further studies considering culture-dependent methods could help to improve our characterization. 

Interestingly, no dairy pathogens such as *Salmonella sp*., *Staphylococcus aureus*, *Listeria monocytogenes* and *Escherichia coli* [4,5] were detected in RMSs nor in PMSs, suggesting that labneh Ambaris may not be a favorable matrix for pathogens growth even when it is made using raw milk. However, other studies have shown the occurrence of contaminated products [17]. Consequently, further studies are needed to elucidate the roles that physicochemical and microbiological compositions might play in ensuring labneh Ambaris safety regarding pathogens.

We herein proposed that many factors, such as the house microbiota, the processing practices, and the presence of microorganisms on utensils and materials possibly affect the microbial diversity of labneh Ambaris. To be able to prove the contributing effect of each of the proposed factors, a large-scale field study could be conducted after our pilot study, in which a larger number of samples would be collected. In addition, studies in controlled laboratory conditions rather than field conditions could be considered. Furthermore, and even if microbial compositions were not influenced by the milk type used, studies should be made to determine if the sensory properties of labneh Ambaris could be affected by pasteurization, which could change the unique qualities of this food product, which is a potential candidate for a Lebanese quality protection label.

In conclusion, our findings revealed that artisanal household milk pasteurization is not a determining factor in structuring the microbial diversity of labneh Ambaris, a traditional fermented milk product from Lebanon. In addition, the microbial compositions of labneh Ambaris samples were highly diverse. However, this variation appears to be minimal or unrelated to whether goat’s milk is artisanally pasteurized or raw.

## Figures and Tables

**Table 1:** Details of the pH values and alpha diversity indices collected for 12 labneh Ambaris samples, 6 made using raw goat’s milk (RMS) and 6 other using artisanally pasteurized goat’s milk (PMS).

Sample Code	Milk Type	pH Values	Bacterial Richness Indices	Bacterial Shannon Indices	Fungal Richness Indices	Fungal Shannon Indices
La-R1	RMS	3.50 ± 0.02	6	1.03	3	0.07
La-R2	RMS	3.01 ± 0.02	12	0.41	2	0.15
La-R3	RMS	3.99 ± 0.02	13	1.51	3	0.66
La-R4	RMS	3.53 ± 0.03	10	1.16	3	0.37
La-R5	RMS	3.58 ± 0.01	10	1.49	3	0.18
La-R6	RMS	3.33 ± 0.02	11	0.38	2	0.16
La-P1	PMS	3.54 ± 0.01	13	1.31	2	0.44
La-P2	PMS	3.73 ± 0.01	13	0.74	4	1.09
La-P3	PMS	3.66 ± 0.01	11	1.4	1	0.00
La-P4	PMS	3.46 ± 0.01	13	0.84	2	0.39
La-P5	PMS	3.66 ± 0.01	12	1.97	2	0.41
La-P6	PMS	3.43 ± 0.01	7	0.78	3	0.20

## Data Availability

The sequencing data generated in this study were deposited in the European Nucleotide Archive (ENA) under accession number PRJEB52591.
